# TNFAIP3 F127C Coding Variation in Greek Primary Sjogren's Syndrome Patients

**DOI:** 10.1155/2018/6923213

**Published:** 2018-12-19

**Authors:** Adrianos Nezos, Eliona Gkioka, Michael Koutsilieris, Michael Voulgarelis, Athanasios G. Tzioufas, Clio P. Mavragani

**Affiliations:** ^1^Department of Physiology, School of Medicine, National and Kapodistrian University of Athens, Athens, Greece; ^2^Department of Pathophysiology, School of Medicine, National and Kapodistrian University of Athens, Athens, Greece; ^3^Joint Academic Rheumatology Program, National and Kapodistrian University of Athens, School of Medicine, Athens, Greece

## Abstract

Tumor necrosis factor, alpha-induced protein 3 (*TNFAIP3*) gene encodes the A20 protein, an important negative feedback regulator of the nuclear factor kappa-light-chain-enhancer of activated B cell (NF-*κ*B) pathway. A coding *TNFAIP3* variant, namely rs2230926, has been previously linked to B cell non-Hodgkin's lymphoma (NHL) development in patients with Sjogren's syndrome (SS) of French and UK origin. Herein, we aimed to determine the prevalence of rs2230926 in a Greek primary SS cohort and explore possible associations with disease characteristics. The rs2230926 gene variant was genotyped in 327 primary Greek SS patients (ninety-one complicated by NHL (SS-lymphoma)) and 448 Greek healthy controls (HC) of similar age and sex distribution. Clinical and laboratory characteristics were also recorded and gene expression of relevant genes of the NF-*κ*B pathway was quantitated by real-time PCR in available whole peripheral blood (PB) from 165 primary SS patients. Increased prevalence of the rs2230926 mutant variant was detected in both SS-lymphoma and SS-nonlymphoma subgroups compared to HC (8.8% vs. 7.6% vs. 3.6%, *p* values: 0.04 and 0.03, respectively) in association with higher IgM, LDH serum levels, and PB *Bcl-XL* transcripts but lower leucocyte and neutrophil counts. Of interest, approximately one-fifth of SS-lymphoma cases with age at disease onset ≤ 40 years carried the rs2230926 variant (18.2% vs. 3.6%, OR 95% (CI): 6.0 (1.8–19.8), *p* value: 0.01). We postulate that deregulation of the NF-*κ*B pathway as a result of the *TNFAIP3* rs2230926 aberration increases SS and SS lymphoma susceptibility particularly in patients with early disease onset.

## 1. Introduction

Primary Sjogren's syndrome (SS)—classically considered a chronic autoimmune exocrinopathy leading to oral and ocular dryness [1–3]—is also characterized by the highest susceptibility for B cell non-Hodgkin's lymphoma (NHL) among all autoimmune diseases [1–5]. Though several clinical, laboratory, and histopathological predictors have been identified over the last decades as reliable predictors for lymphoma development in these patients, the molecular events leading to malignant transformation remain elusive [5–10].

A growing body of evidence suggests that activation of the nuclear factor kappa-light-chain-enhancer of activated B cell (NF-*κ*B) pathway is a critical step in the pathogenesis of both primary SS and B cell hematological malignancies including B cell NHL, the major type of primary SS-related lymphomas. Recent studies revealed that B cell-activating factor-receptor (*BAFF-R*) His159Tyr mutation and a tumor necrosis factor, alpha-induced protein 3 (*TNFAIP3*) germline coding variation—both associated with the activation of the NF-*κ*B pathway—are important contributors in primary SS and SS-related lymphomagenesis [11–16].

The *TNFAIP3* gene encodes the A20 protein essential for the development and functional performance of dendritic, B and T cells and macrophages as well as an important negative feedback regulator of the NF-*κ*B pathway [17]. A20-deficient mice die early due to multiorgan inflammation and cachexia as a result of excessive TNF-induced NF-*κ*B activation [18, 19]. *TNFAIP3* gene variants have been linked to the pathogenesis of both chronic inflammatory and autoimmune disorders [12, 13, 19–22] and B cell lymphomas [23–26].

The current study aims at evaluating the prevalence of the rs2230926 polymorphism in a Greek cohort of primary SS patients and exploring any possible associations with clinical and laboratory SS-related characteristics, B cell NHL development, as well as relevant target genes of the NF-*κ*B pathway.

## 2. Materials and Methods

### 2.1. Study Participants

Peripheral blood was obtained from 327 patients with primary SS (91 complicated by B cell NHL (SS-lymphoma), 236 primary SS without the presence of lymphoma (SS-nonlymphoma)) and 448 healthy individuals (HC) of similar age and sex distribution (Table 1). All primary SS patients were classified according to the 2002 revised version of the European criteria proposed by the American European Consensus Group [27] and followed in the Institute of Systemic Autoimmune and Neurological Diseases (Prof. H. M. Moutsopoulos), Department of Pathophysiology, University of Athens and the Rheumatology Department of the General Hospital of Athens “G. Gennimatas” all located in Athens, Greece. Demographic, clinical, and laboratory features were recorded after thorough chart review. Lymphoma diagnosis in the primary SS-lymphoma group was based on the criteria outlined by the World Health Organization classification [28], and consisted of 72 patients with mucosa-associated lymphoid tissue (MALT) lymphoma and 19 with non-MALT lymphoma (12 diffuse large B cell lymphoma (DLBCL), 4 nodal marginal zone lymphoma (MZL), 2 small lymphocytic lymphoma (SLL), and 1 MALT that was transformed to T cell lymphoma). This study was carried out in accordance with the recommendations of the Ethics Committee of the National and Kapodistrian University of Athens (approved No. 6337) and Laiko General Hospital of Athens Ethics Committee (*ΔΣ*12/20-4-16), with informed consent from all subjects, in accordance with the Declaration of Helsinki. In order to access the ancestry of the study participants, detailed data on family history was collected. Parents and grandparents of 99.4% of the participants were of Greek ethnic heritage, with the rest being of East European descent. None of the study participants had a non-Caucasian ancestry.

### 2.2. Clinical, Serological, and Histopathological Characteristics

Demographics and disease-related clinical and laboratory features were recorded at SS diagnosis after thorough chart review as previously described [29].

### 2.3. DNA Extraction and Genotyping Assay of the *TNFAIP3* rs2230926 Variation

Whole blood samples for DNA extraction were collected in ethylene diamine *tetra*-acetic acid (EDTA) tubes from all study subjects at the time of the enrollment. DNA was isolated from blood samples as previously described [29]. The *TNFAIP3* variation (rs2230926) was identified with real-time PCR by TaqMan SNP Genotyping Assay (Thermo Fisher Scientific, USA). All samples were tested twice, and the results were 100% concordant.

### 2.4. RNA Extraction, cDNA Synthesis, and Quantitation of NF-*κ*B-Related Gene Transcripts

Available peripheral blood samples from 165 SS patients as well as from 20 healthy controls (HC) were collected. Total RNA was extracted and reverse transcribed as previously described [29, 30]. Transcripts related to the NF-*κ*B pathway (*NF-κB1*, *NF-κB2*, *Bcl-2*, and *Bcl-XL*) were quantitated by real time PCR (Table 2), as previously described [31].

### 2.5. Statistical Analysis

The frequency of the rs2230926 polymorphism was determined in the SS and HC groups, and odds ratios (OR) and 95% confidence intervals (CI) adjusted for sex and age were calculated. Demographics and clinical and laboratory features, as well as NF-*κ*B pathway-related gene transcripts, were analyzed for possible associations with the rs2230926 using SPSS v.22.0 and GraphPad Prism. Continuous data were assessed using the Mann–Whitney *U* test. Categorical data were assessed using Fisher's exact test or chi-square accordingly. Results were considered significant when *p* value < 0.05.

## 3. Results

### 3.1. Increased Prevalence of the rs2230926 Polymorphism in SS Compared to HC

**Figure 1:**
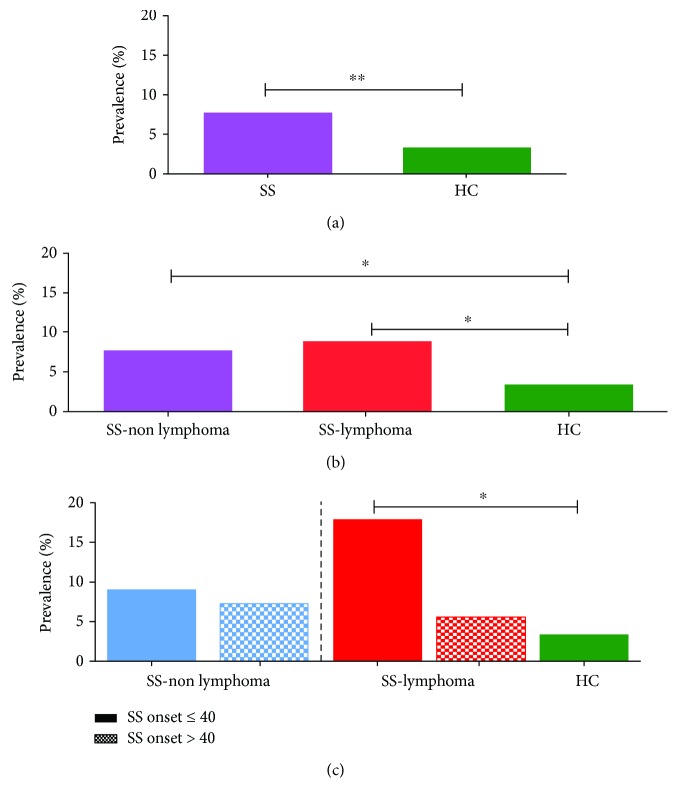
Prevalence of the rs2230926G single-nucleotide polymorphism in primary Sjogren's syndrome (SS) patients and healthy controls (HC). (a) Increased frequency of the rs2230926G variant was detected in all SS study participants compared to HC (26 out of 327 (8.0%) vs. 16 out of 448 (3.6%), *p* value: 0.01, by Fisher's exact test). (b) Both SS-non lymphoma and SS-lymphoma patients displayed higher rates of the rs2230926 compared to HC (18 out of 236 (7.7%) and 8 out of 91 (8.8%) vs. 16 out of 448 (3.6%), *p* values: 0.03 and 0.04, respectively, by Fisher's exact test). (c) When SS subsets were stratified according to the age of SS onset, only the younger-onset (≤40 years) subgroup complicated by lymphoma exhibited significantly higher frequencies of the rs2230926G variant compared to HC (4 out of 22 (18.2%) vs. 16 out of 448 (3.6%), *p* = 0.01, by Fisher's exact test).

As shown in Figure 1(a), significantly increased rates of the rs2230926 mutant variant were observed in the whole primary SS population compared to HC (26 out of 327 (8.0%) versus 16 out of 448 (3.6%); OR 2.3 (95% CI: 1.2–4.4), *p* = 0.01, by Fisher's exact test, adjusted for age and sex distribution). Of note, the variation was found only in heterozygous form in all study participants.

We next estimated the prevalence of the rs2230926 variant in the two distinct SS patient groups compared to HC. Both SS-lymphoma and SS-non lymphoma subsets exhibited higher frequencies of *TNFAIP3* coding variation compared to HC (8.8% (8/91) vs. 7.6% (18/236) vs. 3.6% (16/448), respectively). The calculated OR (95% CI) for the occurrence of SS-lymphoma and SS-non lymphoma in the presence of the rs2230926 variant was 2.6 (1.1–6.3), *p* = 0.04 and 2.2 (1.1–4.2), *p* = 0.03, respectively (Figure 1(b)).

The prevalence of the rs2230926 polymorphism in different lymphoma types was the following: 5/72 (6.9%) in MALT, 1/12 (8.3%) in DLBCL, 1/4 (25%) in MZL, 0/2 (0%) in SLL, and 1/1 (100%) in TCL. Due to the small number of patients, no reliable conclusions can be drawn.

Of interest, when SS subsets were further stratified according to the age of SS onset, only the younger-onset (≤40 years) subgroup complicated by lymphoma exhibited significantly higher frequencies of the rs2230926G variant compared to HC (4 out of 22 (18.2%) versus 16 out of 448 (3.6%), OR (95% CI): 6.0 (1.8–19.8), *p* = 0.01) (Figure 1(c)). Lymphoma was developed after primary SS diagnosis (mean ± SD: 15.8 ± 11.6 years). Three out of four of patients were complicated by MALT and one by DLBCL.

### 3.2. Association of the rs2230926 Polymorphism with Disease-Related Clinical and Laboratory Characteristics

As shown in Table 3, the rs2230926 variation was found to be negatively associated with age at SS diagnosis (45.2 ± 12.9 years in rs2230926 carriers vs. 51.6 ± 13.5 for noncarriers, *p* = 0.02). While no significant associations were detected between the rs2230926 variant and subjective/objective features of oral and ocular dryness or the presence of anti-Ro/SSA and anti-La/SSB antibodies, lower white blood cell and neutrophil counts and higher LDH and IgM levels were detected in patients carrying the rs2230926 variant compared to the non carriers.

### 3.3. The rs2230926 Polymorphism and mRNA Expression of the NF-*κ*B Pathway Target Genes in SS Patients

**Figure 2:**
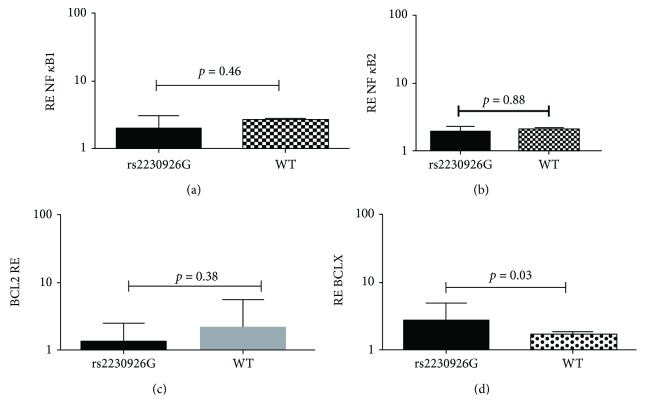
Significantly higher *Bcl-XL* mRNA transcript levels measured by real time-PCR in the whole peripheral blood of SS patients bearing the rs2230926G variant compared to those without the presence of the polymorphism (*p* = 0.03) (d). No statistically significant differences in *NFΚ-B1* (a), *NFκ-B2* (b), and *Bcl-2* (c) gene expression was detected.

In order to explore potential implications of the *TNFAIP3* rs2230926 variation in the NF-*κ*B pathway, we performed mRNA expression analysis for specific genes of the NF-*κ*B pathway in whole peripheral blood RNA samples from primary SS patients carrying or not the mutant variant. As shown in Figure 2(d), we found significantly higher transcript levels of the antiapoptotic gene *Bcl-XL* in rs2230926 carriers, compared to the group without the mutation (2.7 ± 2.1 vs. 1.6 ± 2.0, *p* = 0.03), while *Bcl-2*, *NFκ-B1*, and *NFκ-B2* gene transcripts did not statistically differ between these groups (Figures 2(a)–2(c)).

## 4. Discussion

In the current study, the *TNFAIP3* rs2230926 mutant variant emerged as a risk factor for both primary SS and SS-related lymphoma susceptibility. Following stratification by age at disease onset and lymphoma development, we found that the excess of this variation in the primary SS group compared to HC, was mainly attributed to a heightened prevalence in the early-onset primary SS subset complicated by lymphoma, increasing the risk by 6-fold. Moreover, rs2230926 carriers had lower neutrophil but higher LDH and IgM levels at SS diagnosis, as well as increased peripheral blood expression of the antiapoptotic gene *Bcl-XL* possibly related to NF-*κ*Β pathway activation.

These data are in accord with previously published observations suggesting that *TNFAIP3* rs2230926 coding variation is an additional susceptibility factor for SS [14–16] and SS-related lymphoma [12, 32]. Of interest, the prevalence of the *TNFAIP3* variation seems to be much lower in our HC cohort compared to both UK and French HC cohorts (3.80% vs. 7.14% vs. 12.05%, respectively), implying the presence of a north-south Europe gradient, as previously observed in other autoimmune risk alleles such as the *PTPN22* 1858C>T variant [33].

Activation of the canonical and noncanonical NF-*κ*B pathways in transformed B cells following B cell receptor engagement by exogenous or endogenous antigens as well as CD40L and BAFF ligation have been considered a central event in the pathogenesis of extranodal marginal zone lymphoma of mucosa-associated lymphoid tissue (MALT lymphoma), at various anatomical sites such as salivary glands, stomach, and ocular adnexa. Excessive chronic inflammation either related to microbial infections (such as *Helicobacter pylori*, *Chlamydophila psittaci*, and *Campylobacter jejuni*) or autoantigens in a background of both germline and acquired genetic variations have been previously shown to mediate malignant transformation and lymphoma development (reviewed in [34]).

Of interest, the *TNFAIP3* rs2230926 variant occurred in one-fifth of the patients with younger-onset SS (≤40 years) complicated by lymphoma. Similarly, the His159Tyr mutation of the *BAFF-R* (which leads to noncanonical NF-*κΒ* pathway activation) was found to be present in approximately two-thirds of SS patients with disease onset between 30 to 40 years old with MALT lymphoma [31]. Since data on the presence of the *BAFF-R* His159Tyr mutation were available in the present cohort from our previous study [31], we calculated the frequency of either mutation in the younger-onset SS (≤40 years old) complicated by lymphoma, which was found to be 38.1% vs. 7.2% in the older SS-lymphoma group increasing the risk by 7.9-fold in this population (95% CI: 2.2–28). Taken together, we propose that distinct genetic defects that deregulate both the canonical and the noncanonical NF-*κ*B pathways in young SS patients may accelerate lymphoma development in the setting of SS, possibly through induction of chronic inflammation, B cell stimulation, and survival. In support of the current findings, published data so far, revealed an aggressive disease phenotype and increased risk of lymphoproliferation among SS patients with young disease onset compared to their older counterparts [35], implying the presence of a distinct genetic background in these individuals.

Given that *TNFAIP3* is considered a gatekeeper of the abnormal activation of the NF-*κ*B pathway, the increased frequency of the *TNFAIP3* functional variant in SS patients complicated by lymphoma [12, 32] along with the previously reported lower levels of the A20 protein in MSG tissues derived from SS patients complicated by MALT [11] support the idea of A20-related deregulation of NF-*κ*B pathways in SS-related lymphomagenesis. As a matter of fact, *Bcl-XL* (which belongs in the Bcl-2 antiapoptotic family as a final target of the NF-*κ*B pathway) mRNA transcripts were higher in the SS carriers of the rs2230926G variant compared to SS-noncarrier patients, which may be related to the abnormal activation of the NF-*κ*B pathway due to the *TNFAIP3* variation providing a survival signal to B cells. However, we cannot exclude that other gene variants may also account for this finding. The *Bcl-XL* molecule has been found to play a key role in follicular lymphoma. High *Bcl-XL* levels and low numbers of apoptotic lymphoma cells were reported to be significantly associated with multiple sites of extranodal involvement, elevated lactated hydrogenase level, and short overall survival time [36]. Moreover, *Bcl-XL*-overexpressing mice show enhanced survival of B cells [37] whereas *Bcl-XL*-deficient mice have extensive lymphocyte apoptosis [38].

The strengths of the current study include the complete clinical, serological, and histopathological characteristics of our study participants, the similar age and sex distribution across groups, and the homogeneous Greek Caucasian ancestry (99.4%). However, certain limitations are also recognized. Due to the relatively small number of SS-lymphoma patients, the need for independent validation through multicentric efforts is mandatory. Moreover, more functional experiments are needed to identify the exact role of the rs2230926 A20 genetic variant in SS-related lymphomagenesis. Finally, the presence of other variants in the A20 gene leading to NF-*κ*B pathway activation cannot be excluded.

In conclusion, our data suggest that A20 genetic variants, like rs2230926, may play a central role in the activation of the NF-*κ*B pathway which has been found to be a central pathogenetic event in the malignant transformation in the setting of SS. Elucidation of molecular events leading to SS-related lymphomagenesis will allow eventually the development of tailored therapeutic strategies, opening avenues to personalized medicine approaches.

## Figures and Tables

**Table 1:** Demographics of study participants (age and sex distributions).

Participants	SS patients (*n* = 327)		Healthy controls (*n* = 448)
	SS-nonlymphoma (*n* = 236)	SS-lymphoma (*n* = 91)	
Age (mean years ± SD)	59.5 ± 13.7	59.9 ± 12.5	55.3 ± 18.1
Female sex (%)	93.6%	93.4%	86.0%

**Table 2:** Real-time PCR primers for gene expression quantification.

Full gene name	Gene ID	Accession number	Forward primer	Reverse primer
*Homo sapiens* nuclear factor kappa B subunit 1, mRNA	*NF-κΒ1*	NM_003998.3	CCTGAGACAAATGGGCTACAC	TTTAGGGCTTTGGTTTACACGG
*Homo sapiens* nuclear factor kappa B subunit 2, mRNA	*NF-κΒ2*	NM_001077494.3	GAACTCCTCCATTGTGGAACC	ACCAGCGGTTGAAGCGTTCC
*Homo sapiens* BCL2, apoptosis regulator, mRNA	*Bcl-2*	NM_000633.2	TTGCTTTACGTGGCCTGTTTC	GAAGACCCTGAAGGACAGCCA
*Homo sapiens* B-cell lymphoma-extra large mRNA	*Bcl-XL/Bcl2l1*	NM_138578.2	GCCACTTACCTGAATGACCACC	ACCAGCGGTTGAAGCGTTCC
Glyceraldehyde-3-phosphate dehydrogenase	*GAPDH*	NM_002046	CAACGGATTTGGTCGTATT	GATGGCAACAATATCCACTT

**Table 3:** Clinical and laboratory characteristics of primary Sjogren's syndrome (SS) patients according to the presence of the TNFAIP3 rs2230926 variant.

	TNFAIP3 rs2230926 normal variant (*n* = 301)	TNFAIP3 rs2230926 mutant variant (*n* = 26)	*p* value
Demographics			
Age (mean ± SD years)	59.9 ± 13.7	56.7 ± 9.0	0.24
Sex, female no. (%)	280 (93)	27 (100)	0.39
Age at SS diagnosis (mean ± SD, years)	51.6 ± 13.5	45.2 ± 12.9	0.02
Clinical characteristics			
Focus score (no. of foci/4 mm^2^) (mean ± SD)	2.5 ± 2.3	3.1 ± 2.9	0.72
Tarpley score	2.2 ± 1.1	2.5 ± 1.1	0.30
Erythrocyte sedimentation rate (ESR) (mm/h)	39 ± 26	36 ± 27	0.77
Dry mouth (%)	278 (92.4)	25 (96.2)	0.70
Dry eyes (%)	275 (91.4)	25 (96.2)	0.71
Salivary gland enlargement (SGE) (%)	108 (35.9)	6 (23.1)	0.21
Raynaud's syndrome (%)	85 (28.3)	8 (30.8)	0.82
Lymphadenopathy (%)	70 (23.3)	6 (23.1)	1.00
Splenomegaly (%)	11 (3.7)	1 (3.8)	1.00
Parpable purpura (%)	60 (19.9)	8 (30.8)	0.21
Arthritis (%)	79 (26.2)	5 (19.2)	0.49
Arthralgias/myalgias (%)	213 (70.8)	19 (73.1)	1.00
Interstitial lung disease (%)	24 (8.0)	4 (16.0)	0.25
Liver involvement (autoimmune cholangitis) (%)	15 (5.0)	1 (3.8)	1.00
Kidney involvement (nephrocalcinosis) (%)	4 (1.3)	1 (3.8)	0.34
Lymphoma (%)	82 (27.3)	8 (30.8)	0.82
Laboratory characteristics			
Whole salivary flow (ml/15 min) (mean ± SD)	1.6 ± 1.2	1.3 ± 1.3	0.43
Presence of anti-Ro/SSA autoantibodies (%)	223 (75.3)	22 (88.0)	0.22
Presence of anti-La/SSB autoantibodies (%)	123 (41.6)	11 (44.0)	0.84
Positive rheumatoid factor (%)	173 (62.9)	14 (58.3)	0.67
Low-complement component 4 (C4) levels (<20 mg/dl), no (%)	178 (60.5)	15 (62.5)	0.82
Peripheral blood leucocyte count (mean ± SD per mm^3^)	1629.5 ± 669.2	1566.0 ± 845.1	0.44
Peripheral blood neutrophil count (mean ± SD per mm^3^)	3464.7 ± 1705.8	2690.4 ± 628.3	0.009
Peripheral blood monocyte count (mean ± SD per mm^3^)	424.0 ± 228.0	345.9 ± 165.4	0.18
White blood cell count (mean ± SD per mm^3^)	5682.0 ± 1924.3	4915.8 ± 1125.7	0.04
Gamma globulin levels (mean ± SD)	33.9 ± 26.2	35.9 ± 27.1	0.72
Lactate dehydrogenase (LDH) (mean ± SD) levels	284.6 ± 122.1	518.2 ± 238.9	0.005
β2-Microglobulin (mean ± SD)	4.0 ± 2.6	5.6 ± 3.5	0.26
Immunoglobulin IgA (mean ± SD)	299.8 ± 146.7	403.3 ± 194.8	0.21
Immunoglobulin IgG (mean ± SD)	1683.5 ± 690.0	2790.0 ± 1656.7	0.08
Immunoglobulin IgM (mean ± SD)	183.1 ± 125.6	339.8 ± 308.1	0.02
Presence of monoclonal band (%)	34 (12.0)	2 (8.0)	0.75
Cryoglobulins (%)	35 (14.1)	4 (25.0)	0.27

## Data Availability

All data used to support the findings of this study are included within the article.
